# Relationship between liver and cardiometabolic health in type 1 diabetes

**DOI:** 10.3389/fendo.2024.1505430

**Published:** 2024-11-13

**Authors:** Emir Tas, Bach-Mai Katherine Vu, Brenda Mendizabal, Ingrid Libman, Radhika Muzumdar

**Affiliations:** ^1^ Division of Endocrinology and Diabetes, University of Pittsburgh Medical Center (UPMC) Children’s Hospital of Pittsburgh, Pittsburgh, PA, United States; ^2^ Department of Pediatrics, University of Pittsburgh, Pittsburgh, PA, United States; ^3^ Division of Cardiology, University of Pittsburgh Medical Center (UPMC) Children’s Hospital of Pittsburgh, Pittsburgh, PA, United States

**Keywords:** hepatosteatosis, cardiovascular disease, insulin resistance, inflammation, type 1 diabetes

## Abstract

**Introduction:**

Type 1 diabetes (T1D) is a chronic condition marked by insulin deficiency and hyperglycemia, with an increasing global incidence, particularly among children. Despite improvements in diabetes management, individuals with T1D continue to experience higher rates of cardiovascular disease (CVD), the leading cause of mortality in this population. Traditional CVD risk factors such as dyslipidemia and poor glycemic control are insufficient to fully explain the elevated risk in T1D, prompting further investigation into additional factors. Emerging evidence suggests that metabolic dysfunction-associated steatotic liver disease (MASLD) plays a critical role in this heightened CVD risk.

**Objective:**

This narrative review aims to explore the relationship between MASLD and CVD in individuals with T1D. The review focuses on the prevalence of MASLD, its contributing risk factors, and the potential impact of liver dysfunction on cardiovascular outcomes in this population.

**Methods:**

A review of existing literature was conducted, focusing on observational studies, cohort studies, and meta-analyses that investigate the prevalence of MASLD in T1D populations and its association with CVD. The review also examines the physiological mechanisms linking MASLD and CVD, including insulin resistance, systemic inflammation, and hepatic dyslipidemia. Key studies were evaluated to identify patterns in MASLD prevalence based on diagnostic modalities and to assess the independent contribution of MASLD to cardiovascular risk in T1D patients.

**Conclusion:**

MASLD is increasingly recognized as a significant contributor to CVD in individuals with T1D, particularly in those with shared risk factors like obesity and insulin resistance. Evidence suggests that MASLD exacerbates hepatic and systemic metabolic dysfunction, increasing CVD risk through mechanisms such as chronic inflammation and atherogenic lipid profiles. Routine liver health assessments and tailored management strategies targeting MASLD should be incorporated into clinical care for individuals with T1D to mitigate long-term cardiovascular complications.

## 1 Introduction

Type 1 diabetes (T1D) is a common chronic metabolic disorder characterized by insulin deficiency and hyperglycemia, with an estimated worldwide prevalence of 95 per 100,000 people (1). Globally, the incidence of T1D is rising, particularly among children, although an estimated 25-50% of new diagnoses occur in adults (2–5). Managing T1D poses significant health challenges due to the chronic and highly demanding nature of the disease. Despite meticulous management, individuals with T1D remain at risk of developing both acute and long-term complications.

Recent advances in treatment, particularly hybrid closed-loop systems, have substantially improved quality of life and reduced the incidence of insulin-related acute complications such as severe hypoglycemia, hyperglycemia, and ketoacidosis (6). However, despite significant advances in diabetes management and recommendations of aggressive strategies to control cardiometabolic risk factors (i.e., lower blood pressure and cholesterol thresholds), individuals with T1D continue to experience higher rates of morbidity and mortality compared to the general population (7). Cardiovascular diseases (CVD) remain the leading cause of death among people with T1D. While traditional risk factors such as dyslipidemia, hypertension, and poor glycemic control play a role, they do not fully explain the increased risk of adverse CVD outcomes. This underscores the need for further research to identify additional contributing factors (8).

Recent studies suggest a link between metabolic dysfunction–associated steatotic liver disease (MASLD) and CVD In patients with T1D. In this narrative review, we will explore risk factors for CVD, the emerging role of the liver in cardiometabolic health, and management of MASLD and CVD risk in individuals with T1D. A deeper understanding of MASLD’s impact on CVD in T1D could lead to more accurate risk assessments and improved prevention strategies.

## 2 Cardiovascular disease in type 1 diabetes

Cardiovascular disease is highly prevalent among individuals with T1D and is the leading cause of morbidity and mortality in this population (7). Coronary artery disease/atherosclerosis is the most common type of CVD (9). However, peripheral artery disease, cerebrovascular disease (stroke), heart failure, and cardiomyopathy also occur more frequently in those with T1D compared to the general population.

The increased burden of CVD in individuals with T1D has been consistently reported in observational studies across various cohorts. Earlier studies in individuals with juvenile diabetes (diagnosed before age 21 years) reported higher cumulative mortality rate (35 ± 5%) due to coronary artery disease compared to healthy subjects in the Framingham Heart Study (8% for men and 4% for women) by age 55 (10). Similarly, the Pittsburgh Epidemiology of Diabetes Complications study of childhood-onset type 1 diabetes showed a 19-fold increased relative risk of CVD mortality particularly in younger adults (< 45 years) compared to the age-matched background population (11). More recently, in a large nationwide cohort study in Denmark involving young people (ages 1–39), CVD mortality was found to be 11 times higher in individuals with T1D compared to age-matched individuals without diabetes (12).

Among adults with T1D, the prevalence of CVD is seven times higher than in healthy controls and typically arises a decade earlier, resulting in a shortened life expectancy by an average of 10 years (7, 13). This was further supported by a Swedish cohort study, which demonstrated that the age at T1D diagnosis is an important determinant of CVD risk. Individuals diagnosed between ages 0–10 had a fivefold higher risk of developing CVD compared to those diagnosed between ages 26–30 (14). Additionally, early-onset T1D was associated with a greater loss of life expectancy compared to late-onset T1D, with CVD-related mortality being higher among women than men (14).

Although clinically manifest CVD is rare before adulthood, early signs – such as increased arterial intima-media thickness (IMT), arterial stiffness, and decreased pulse wave velocity (PWV) – can be detected during childhood. These findings are not surprising, as CVD risk factors are prevalent among youth with T1D. In a cohort study involving multi-ethnic children with T1D in the UK, up to 60% of patients were found to have at least one traditional CVD risk factor (15). Several studies, including SEARCH-CVD (an ancillary study to SEARCH for Diabetes in Youth) in the US, have shown increased carotid intima-media thickness (cIMT) in youth with T1D compared to healthy controls (16). A systematic review and meta-analysis by Giannopoulou et al., which compiled data from 23 studies (n=2,860 T1D and n=1,861 control subjects), found higher cIMT and PWV in children with T1D than in matched controls (17).

## 3 CVD risk factors in individuals with diabetes

The increased prevalence of CVD in individuals with diabetes is attributed to a combination of modifiable and non-modifiable factors, further complicated by social determinants of health and individual-level differences in behaviors (18). Prolonged hyperglycemia, dyslipidemia, hypertension, smoking, obesity, and presence of microvascular complications (i.e., nephropathy) are regarded as traditional risk factors for adverse CVD outcomes (19). While improvements in these risk factors are associated with reductions in cardiovascular events and decreased mortality, even sustained improvements do not fully mitigate adverse CVD outcomes. This suggests the role of additional unexplored risk factors in CVD morbidity and mortality in T1D (20). Recently, emerging roles of insulin resistance (IR), visceral adiposity, chronic inflammation, increased oxidative stress, epigenetic modification, and cardiac autoimmunity have been recognized in the pathogenesis of CVD in diabetes (21, 22).

## 4 The link between metabolic dysfunction-associated steatotic liver disease, CVD, and diabetes

### 4.1 MASLD and diabetes

Metabolic dysfunction–associated steatotic liver disease (MASLD), formerly known as nonalcoholic fatty liver disease or NAFLD, is the most common etiology of chronic liver disease in children and adults (23, 24). The MASLD spectrum ranges from simple hepatosteatosis (HS) to metabolic dysfunction–associated steatohepatitis (MASH), cirrhosis, and eventually liver failure. MASLD is considered the hepatic manifestation of metabolic syndrome and is strongly associated with obesity, insulin resistance, dyslipidemia, hypertension, and increased waist circumference. It is estimated that up to 10% of all children in the United States have some form of steatotic liver disease, with a prevalence of up to 50% among girls with obesity and polycystic ovarian syndrome (PCOS) (23, 25). MASH-related cirrhosis is one of the leading causes of liver transplantation (25). Despite the well-established relationship between MASLD and T2D, the association between MASLD and T1D has been largely unexplored.

Rates of obesity among individuals with T1D are increasing in parallel with global population trends in both children and adults, with more than a third of patients with T1D estimated to have overweight or obesity (26, 27). Excess weight gain may also occur over time due to intensive insulin therapy, inadequate exercise, and excess food intake, often in defense against hypoglycemia. Despite a substantial proportion of T1D subjects sharing similar phenotypes to those with T2D, such as obesity, insulin resistance, dyslipidemia, chronic hyperglycemia, and systemic hyperinsulinemia, routine screening for MASLD in T1D is not the standard of care in clinical practice. However, ADA’s 2024 standards of care guidelines urge the providers to consider screening for fibrosis in people with T1D in the presence of additional risk factors for MASLD such as obesity, incidental hepatic steatosis on imaging, or elevated plasma aminotransferases.

### 4.2 Prevalence of MASLD in T1D

Epidemiological studies assessing the prevalence of MASLD in youth with T1D are scarce. Furthermore, results from the available studies are not readily comparable due to differences in the diagnostic modalities and study populations (**Table 1**). In a meta-analysis assessing MASLD prevalence in T1D, comprising 20 studies in children and adults, De Vries and colleagues (28) found a pooled prevalence of 7.9% (95% CI: 2.6-15.5%) among children with T1D (n=202 in 3 studies), compared to 22.0% (95% CI: 13.9-31.2%) among adults with T1D (n=3699 in 17 studies).

**Table 1:** MASLD prevalence studies in pediatric T1D patients.

Reference (#)	N	Sex (Male %)	Age (years)	Diabetes Duration (years)	HbA1c (%)	Diagnostic Tool for MASLD	MASLD Prevalence (%)	Key features of the study
**Al-Hussaini** (29)	106	42	8.5 ± 2.8	2.2 ± 2.1	10.7 ± 2.4	Ultrasound	21	Half of the patients with abnormal US had hepatomegaly
**El-Karaksy** (30)	692	48	9.7 ± 4.2	6.9 ± 3.7	7.7 ± 1.6	Hepatomegaly on exam, elevated liver enzymes, ultrasound	8.7	Patients with viral hepatitis (n=25) were included in the analysis.
**Elkabbany** (31)	100	39	13.7 ± 1.9	6.5 ± 1.7	9.7 ± 2.4	Hepatomegaly on exam, elevated liver enzymes, ultrasound, transient elastrogram	12	Patients with autoimmune hepatitis and viral hepatitis were included in the analysis but MASLD prevalence given separately.
**Aydin** (32)	110	47	12.5 ± 2.8	3.1 ± 2.2	11.4 ± 2.1	Ultrasound	15.5	Patients with abnormal US had HbA1c >9%
**Abdallah** (33)	74	50	14.3 ± 3.0	6.3 ± 3.0	10.3 ± 2.0	Ultrasound	62	Only 6.5% of patients with abnormal US had elevated liver enzymes.
**Regnell** (34)	22	54	13.5 (range: 9-17)	5.9 (range: 0–13)	7.9 (range: 6.4–11.1)	MRI	0	There were none to a few overweight or obese subjects in this cohort.
**Kummer** (35)	93	49	11.9 ± 4.0	4.6 ± 3.5	7.6 ± 0.8	Liver Enzyme, Ultrasound,	10.8	Only two patients had obesity and only 1 had ALT level twice the upper limit of normal.
**Sae-Wong** (36)	50	44	16.9 (IQR: 13.6-20)	6.5 (IQR: 4-11)	8.7 (IQR: 7.9-10.1)	MRI	10	Of the 5 patients with MASLD, 2 were overweight and 2 were obese.
**Tas** (37)	49	53	14.5 ± 3	4.9 ± 3.6	8.7 ± 2	Transient elastrogram	33	A CAP >241 dB/m was used for MASLD diagnosis. 30% of the T1D patients had obesity.
**Koutny** (39)	32,325	53	Average age across strata ranges between 11.3 and 15 yrs	Average across strata ranges between 1.6 and 4.7 years	Groups stratified per HbA1c (<9%, 9-11%, >11%)	Liver enzyme	14.4	For male participants, ALT levels > 26 U/L and for female participants> 22 U/L were considered elevated

MASLD prevalence has consistently been reported as higher in studies utilizing ultrasound as the diagnostic tool (29–33). This may be partly due to ultrasound’s inability to distinguish glycogenopathy and fatty infiltration in liver images. For example, Regnell et al. (34) found no cases of MASLD using hepatic fat fraction MRI for diagnosis in a small set of children (n=22) with T1D. Similarly, Kummer et al. (35) reported only one potential MASLD case in a cohort of 93 children using a combination of ultrasound, transient elastography (FibroScan^®^), and acoustic radiation force imaging. However, these studies had very few or no subjects with obesity, limiting their generalizability given the rising prevalence of obesity among children with T1D. In contrast, a study using MRI as the diagnostic tool linked MASLD to obesity (36). Likewise, in our previous work, we demonstrated that pediatric patients with T1D and obesity (n=15) shared similar clinical, laboratory, and imaging findings (FibroScan^®^) compared to patients with obesity without T1D (n=28), supporting the notion that obesity is the major determinant of MASLD (37). In a cross-sectional study, West et al. (38) demonstrated that elevated serum alanine aminotransferase (ALT) levels were more common in T1D patients (n=517, majority adults) than in the general population, with a prevalence of 9.5% (95%CI: 7.1-12.3%).

More recently, Koutny et al., using serum ALT levels as a proxy for MASLD, examined the longitudinal data from 32,325 children (age 2-17 years) with T1D in the Diabetes-Patienten-Verlaufsdokumentation study (39). They reported that children with poorly controlled T1D (i.e., HbA1c > 11%) had 2.54 (95% CI: 2.10-3.10) times the odds of having elevated liver enzymes after adjusting for sex, age, diabetes duration, and overweight status. Their findings suggest that the association between T1D and MASLD is independent of weight status.

Estimates of MASLD burden among T1D patients vary widely, depending on the population studied and diagnostic modality used, leaving the true prevalence and impact of MASLD in T1D remain unclear (40). Moreover, there is a paucity of pediatric studies in the T1D population utilizing histology (i.e., liver biopsy) as the diagnostic tool. As a result, the prognostic importance of elevated liver enzymes or abnormal imaging findings remains unknown. In a more recent meta-analysis, Ciardullo and Perseghin reported a 5% prevalence for increased liver stiffness, a surrogate marker for fibrosis, in adult subjects with T1D (41).

### 4.3 Factors contributing to MASLD development in T1D

The “two-hit” model and its extension, the “multiple parallel hits” hypothesis, offer a detailed understanding of the pathogenesis of MASLD and its systemic repercussions. According to these frameworks, chronic inflammation in MASLD emerges from a combination of various stressors acting simultaneously, such as insulin resistance and lipotoxicity, particularly in genetically susceptible individuals (42, 43). Excessive liver fat accumulation in MASLD results from an imbalance between lipid accumulation and utilization. This imbalance is driven by peripheral and hepatic IR, which promotes increased hepatic uptake of free fatty acids and enhanced triglyceride synthesis. When the liver’s ability to oxidize fatty acids or export triglycerides as very-low-density lipoproteins cannot keep pace with fat accumulation, hepatosteatosis occurs.

In T1D, several factors contribute to MASLD, mirroring those seen in obesity and T2D. These factors include poor glycemic control, obesity, IR, dyslipidemia, lipoprotein abnormalities, poor dietary habits, impaired systemic to portal insulin gradient, and altered gut microbiome in genetically susceptible individuals (44, 45).

In a cross-sectional study of young adults with T1D (n=659, mean age 37 ± 13 years, mean diabetes duration 20 ± 12 years), Della Pepa et al. (46) reported an association between elevated HbA1c and MASLD. In their cohort, patients with HbA1c > 7.6% had significantly higher liver indices—the fatty liver index (FLI) and hepatic steatosis index, two commonly used non-invasive composite scoring systems to determine risk for MASLD with higher scores predicting MASLD. This association was independent of obesity status. A more recent cross-sectional study examined the association of continuous glucose meter-derived outcomes with MASLD in adults with T1D (n=302, median age 49 [34-61] years, median diabetes duration 29 [17-38] years).The study found independent associations between MASLD (ultrasound diagnosed) and time in range (55% ± 16%, p=0.028), time above range (p=0.007), and time below range (p=0.036) (47). These studies indicate that overall glycemic control and blood glucose variability are linked to MASLD.

As mentioned above, individuals with T1D often have obesity rates comparable to their age-matched peers at diagnosis and are at risk for further weight gain due to systemic insulin therapy, frequent snacking to manage hypoglycemia, and reduced physical activity (48–50). The interplay between hyperglycemia, hyperinsulinemia, and hyperglucagonemia exacerbates IR. Elevated glucose levels enhance hepatic glucose uptake through glucose transporter 2 (GLUT 2) and stimulate genes encoding sterol regulatory element-binding proteins (SREBP) and carbohydrate-responsive element-binding protein (ChREBP), two transcription factors upregulating hepatic *de novo* lipogenesis (51). Additionally, systemic hyperinsulinemia and reduced hepatic insulin clearance—potentially due to decreased expression of carcinoembryonic antigen-related cell adhesion molecule 1 (CEACAM1) because of hepatic IR—further promote hepatic lipogenesis (52).

These mechanisms collectively contribute to the development of MASLD in T1D patients. Moreover, steatotic or inflamed liver releases pro-inflammatory and pro-coagulant factors mediating vascular endothelial damage (53).

### 4.4 Does MASLD increase CVD risk?

The link between MASLD and adverse CVD outcomes is well-documented in subjects with obesity and T2D (54). Examining the nationwide health screening database of more than 9.5 million adults (age 40-64 years) in South Korea, Lee et al. (55) estimated a hazard ratio of 1.43 (95% CI: 1.41-1.45) for CVD events – such as myocardial infarction, ischemic stroke, heart failure, or CVD-related death – in individuals with MASLD compared to a reference group with no fatty liver disease (unadjusted for diabetes or other potential mediators). Similarly, Vaz et al. (56) analyzed the data obtained longitudinally from adults in Australia (25,469 person-years follow-up) and found that subjects with MASLD had a 1.5 times (95% CI: 1.11-2.06) the hazard ratio of adverse CVD outcomes, including non-fatal myocardial infarction, cerebrovascular accidents, and CVD-related deaths, after adjusting for demographic covariates and known cardiometabolic risk factors, including diabetes. Both studies used fatty liver index, a biomarker panel for MASLD diagnosis.

The relationship between MASLD and CVD is hypothesized to be bidirectional such that MASLD is not only a marker of CVD but also a contributor to its pathogenesis. Hepatosteatosis might contribute to hepatic and systemic IR, and atherogenic dyslipidemia (57). Steatosis triggers inflammatory pathways and cytokine release, leading to liver fibrosis and systemic inflammation. Studies highlight the predictive role highly sensitive C-reactive protein as a marker of low-grade systemic inflammation in adverse CVD outcomes in patients with MASLD (58, 59). However, due to overlapping risk factors for the development of CVD and MASLD, it remains challenging to assess a causal relationship (**Table 2**).

**Table 2:** Risk factors for CVD and MASLD in type 1 diabetes and diagnostic tools.

Traditional Risk Factors	Emerging Risk Factors
Obesity	Chronic inflammation
Hyperglycemia (i.e., poor glycemic control)	Oxidative stress
Dyslipidemia	Gut microbiota
Hypertension	Environmental factors
Smoking	Cardiac autoimmunity^$^
Lifestyle factors (unhealthy diet, limited physical activity)	Microvascular complications of diabetes
Insulin resistance	^$^Unique to CVD.
Visceral adiposity (i.e., increased waist circumference)	
Screening/Diagnosis of MASLD	
Physical examination (i.e., hepatomegaly*)	
Increased liver enzymes (ALT, AST, GGT)^,*	
Ultrasound (B-mode)^	
Vibration controlled transient elastogram (i.e., Fibroscan^®^)	
Magnetic resonance elastogram (i.e., spectroscopy, PDFF) ^#^	
Liver biopsy^##^	

* Could indicate hepatic glycogenopathy.

^ Low specificity in early stages/low degree of hepatosteatosis.

^#^ Non-invasive reference standard tool.

^##^ Gold Standard. Allows histological grading.

^$^ refers that “cardiac immunity” is a unique risk factor for CVD and it does not apply to MASLD.

Emerging evidence suggests a connection between MASLD and CVD in adolescents and young adults. A Swedish nationwide cohort study (60) found that children and young adults under 25 years (n=699) with biopsy-confirmed MASLD had significantly higher rates of incident CVD events compared to matched population controls. Specifically, they reported adjusted hazard ratios (aHR) of 2.33 (95% CI: 1.43 to 3.78) for ischemic heart disease, 3.07 (95% CI: 1.62 to 5.83) for congestive heart disease, and 3.16 (95% CI: 1.49 to 6.68) for arrhythmia after a median follow-up of 16.6 years. Notably, the incident rates were higher in the advanced liver disease subgroup, suggesting a possible role of the liver in the progression of CVD. Additionally, a recent systematic review and meta-analysis (61) that examined the data from 4 large cohorts (10,668,189 participants) corroborated these findings, demonstrating a strong link between MASLD in young adults and children and the risk of CVD, with a HR of 1.63 (95% CI: 1.46–1.82, p<0.001).

### 4.5 MASLD and CVD in patients with T1D

Data on CVD and MASLD in T1D population is scarce. Major barriers to directly assessing this relationship, particularly in children and young adults, include the natural course of MASLD and CVD (i.e., long asymptomatic phases), the lack of non-invasive diagnostic tools to stage MASLD, the reliance on composite scores rather than clinical outcomes in CVD assessment, and the presence of confounding factors that often co-exist with both conditions such as dyslipidemia, hypertension, visceral adiposity, and obesity.

Despite these challenges, a few studies have shown that MASLD is independently associated with adverse cardiovascular outcomes (62) and microvascular complications (63, 64) in individuals with T1D. In an observational study of 286 adults with T1D (median diabetes duration 17 [10-30] years), Mantovani et al. (62) assessed baseline MASLD status via ultrasound and followed the patients for a mean of 5.3 ± 2.1 years for the occurrence of incident CVD events. After adjusting for demographic and clinical covariates, the incidence of composite CVD events was found to be 6.73 times (95% CI: 1.2–38.1) higher in those with MASLD than those without MASLD, highlighting the importance of non-traditional risk factors for adverse CVD outcomes. The same research group replicated their findings on two other cohorts, demonstrated an independent relationship between MASLD and CVD, even after adjusting for age, sex, body mass index, glycemic control (i.e., HbA1c), diabetes duration, plasma lipids, albuminuria, smoking status, and family history of CVD (65, 66). It is important to note that the presence of fatty liver was assessed using ultrasound, and the prevalence of MASLD varied between 44 and 52% in these studies, which is significantly higher than most other prevalence studies (28).

Cross-sectional studies from two different research groups reported similar findings. Zhang et al. (67) assessed the relationship between MASLD and cIMT in adult T1D patients. Of the 722 patients (mean age 46 ± 13 years, diabetes duration 7.5 ± 4.2 years), 15.9% had MASLD by ultrasound. This subgroup had greater cIMT (0.81 ± 0.25 vs. 0.69 ± 0.18 mm; p<0.001) than non-MASLD patients even after adjusting for potential confounders. Moreover, MASLD was independently associated with cIMT in a linear regression model (standardized β, 0.151, p < 0.001). Similarly, Serra-Planas et al. (68) examined the prevalence of MASLD and its relationship to CVD risk factors in 100 adult T1D patients (mean age 39.4 ± 7.8 years, diabetes duration 21.7 ± 8.6 years. They reported a lower MASLD (only 12% of the cohort) by ultrasound. Those with MASLD had a greater cIMT than those without NAFLD (0.65 ± 0.17 vs 0.55 ± 0.14 mm; p=0.029), but the groups did not differ regarding carotid artery calcification score or the presence of carotid plaque—two other markers of CVD.

In a recently published observational prospective study with a median follow-up of 11 years, Garofolo et al. (69) examined the association between MASLD (assessed by FLI), all-cause mortality, and first cardiovascular events in 774 young adult T1D patients (mean age 30.3 ± 11.1 years, diabetes duration 18.5 ± 11.6 years, HbA1c 7.8 ± 1.2%). They showed an increased incident of CVD events in those with elevated FLI, even after adjusting for clinical and biochemical factors considered important in Steno Type 1 and EURODIAB Risk Engines for determining CVD risk.

Traditionally, CVD risk assessment in T1D has focused on factors like glycemic control, blood pressure, lipid levels, and prevention and treatment of diabetic kidney disease. Although the aforementioned studies show an independent association between MASLD and CVD in T1D population, evidence providing a causal relationship is lacking. Given the accumulating data suggesting that MASLD is an independent CVD risk factor, the liver health assessments should be included in risk estimation and individualized treatment.

In an ongoing clinical trial in adults at the University Hospital, Antwerp, Belgium (NCT04664036), the researchers are examining the utility of non-invasive diagnostic tools for assessing MASLD and their correlation with microvascular and macrovascular complications of diabetes in a prospective manner. The findings of study are expected to shed light on liver’s role in the development of CVD in individuals with T1D. Detecting and managing MASLD could potentially reduce the risk of developing cardiovascular complications, the leading cause of morbidity and mortality in individuals with T1D.

## 5 Conclusions

In conclusion, the complex interplay between MASLD and CVD in T1D patients underscores the importance of early detection and comprehensive management of MASLD. Despite advancements in T1D therapies, including continuous glucose monitors and hybrid closed-loop systems, individuals with T1D continue to face an elevated risk of CVD, which is not fully explained by traditional factors such as dyslipidemia, hypertension, and poor glycemic control. Emerging evidence now points to MASLD as a significant contributor to cardiovascular risk, even in the T1D population. MASLD, once primarily linked to T2D, is increasingly recognized in T1D due to overlapping risk factors such as obesity, insulin resistance, and chronic inflammation. This bidirectional relationship between MASLD and CVD, characterized by hepatic insulin resistance, atherogenic dyslipidemia, and systemic inflammation, suggests that MASLD may be both a marker and a mediator of CVD risk in T1D.

Considering available and proposed treatment options, managing MASLD in T1D patients requires a multifaceted approach. Lifestyle interventions, including weight loss, exercise, and dietary modifications, remain central to MASLD management, particularly in individuals with concurrent obesity. Pharmacological therapies targeting the liver are also being explored. Current treatments under investigation for MASLD in T2D, such as GLP-1 receptor agonists (70) and SGLT-2 inhibitors (71), have shown promise in improving liver steatosis and reducing CVD risk and may hold potential for T1D patients. These agents not only improve glycemic control but also promote weight loss, reduce inflammation, and improve lipid profiles—key factors in managing MASLD. Additionally, new therapies targeting fibrosis, such as FGF21 analogs, are in clinical trials and may offer benefits in preventing progression from simple steatosis to more advanced liver disease (72). While no specific treatments have been validated for MASLD in T1D, ongoing research in T2D could inform future therapeutic strategies. Early liver health assessments and tailored interventions in T1D patients could significantly reduce the burden of CVD, emphasizing the importance of integrated cardiometabolic care for improving long-term outcomes.
